# Non-random associations in group housed rats (*Rattus norvegicus*)

**DOI:** 10.1038/s41598-021-94608-4

**Published:** 2021-07-28

**Authors:** Leanne Proops, Camille A. Troisi, Tanja K. Kleinhappel, Teresa Romero

**Affiliations:** 1 Department of Psychology, Centre for Comparative and Evolutionary Psychology, University of Portsmouth, Portsmouth, UK; 2 School of Life Sciences, College of Sciences, University of Lincoln, Lincoln, UK; 3 Department of Experimental Psychology, Ghent University, Ghent, Belgium

**Keywords:** Animal behaviour, Social behaviour

## Abstract

Ecological factors, such as predation, have traditionally been used to explain sociability. However, it is increasingly recognised that individuals within a group do not associate randomly, and that these non-random associations can generate fitness advantages. The majority of the empirical evidence on differentiated associations in group-living mammals, however, comes from a limited number of taxa and we still know very little about their occurrence and characteristics in some highly social species, such as rats (*Rattus *spp.). Here, using network analysis, we quantified association patterns in four groups of male fancy rats. We found that the associations between rats were not randomly distributed and that most individuals had significantly more preferred/avoided associates than expected by random. We also found that these preferences can be stable over time, and that they were not influenced by individuals’ rank position in the dominance hierarchy. Our findings are consistent with work in other mammals, but contrast with the limited evidence available for other rat strains. While further studies in groups with different demographic composition are warranted to confirm our findings, the occurrence of differentiated associations in all male groups of rats have important implications for the management and welfare of captive rat populations.

## Introduction

Sociability has traditionally been explained in terms of ecological factors, such as predation and resource distribution^1,2^. While these factors may explain the occurrence and diversity of social systems, it is increasingly recognised that individuals within a group do not associate randomly, and that the formation of these non-random associations can generate strong fitness advantages in terms of survival and reproductive performance^3^. The impact of differentiated associations on individuals’ fitness extends beyond the benefits derived from associations within the reproductive context^4^. For example, forming strong well differentiated associations among same-sex individuals in baboons (*Papio hamadryas ursinus*)^5^, horses (*Equus caballus*)^6^, and house mice (*Mus musculus*)^7^ affects individuals’ long-term reproductive success and longevity, with individuals forming the strongest and most stable associations reproducing more successfully and living longer than other animals. More recent research suggests that the benefits of sociality may be more complex than previously described, with fitness related benefits associated with the number of associations (strong or otherwise) individuals have in their social group^8,9^.

Following Hinde’s conceptual framework^10^, social relationships are considered to be the outcome of a series of social interactions between individuals and can be characterised by their content (e.g., sexual, parenting, affiliation or conflict), quality (e.g., intensity of affiliation or aggression), and temporal patterning (e.g., frequency)^10^. According to this framework, social relationships might vary among dyads since an individual can interact with different individuals in different ways. Differentiated social relationships occur when this variation of interaction patterns show consistency over time. When individuals are more likely to interact or associate with one another than with other potential partners, individuals can be said to have preferred social partners^11,12^.

Animals can develop social preferences for particular group mates depending on a number of factors. Close associations between individuals are often strongly correlated with their genetic relatedness (e.g., primates^13^; ring-tailed coatis (*Nausa nasua*)^14^, giraffe (*Giraffa camelopardalis*)^11^), but preferred associations are not limited to kin (e.g., equids^6^; greater ani (*Crotophaga major*)^15^). In many mammalian species sex also influences affiliation patterns among individuals in a group, with females affiliating with each other at high rates in, for instance, many cercopithecine species^16^. Social preferences might also emerge as a by-product of an attraction to high-ranking individuals who might provide benefits to lower-ranking animals such as tolerance at feeding sites or support in alliances^13^. Other variables such as similarity in age (mouflons (*Ovis gmelini*)^17^; coatis^14^) or personality traits (brown capuchin monkeys (*Sapajus spp.*)^18^) have also been identified as factors predicting partner choice.

Despite the apparent ubiquitous occurrence of differentiated associations in group-living mammals and their potential impact on individuals’ fitness, the vast majority of empirical evidence comes from a limited number of taxa (mainly primate species) and we still know very little about their occurrence and characteristics in some highly social species, such as rats (*Rattus spp.*). In the wild, rats form complex societies of up to several hundred individuals, often consisting of sub-groups of females and males with overlapping home ranges^19^. In domestic and laboratory settings, however, they are rarely housed in groups larger than pairs, offering little opportunity to fully express the range of social strategies that are part of their natural repertoire. Thus, despite 150 years of captive breeding and estimates suggesting there are over twenty million rats in laboratories across the world^20^, we still know surprisingly little about their natural social behaviour or organisation. Most of the data on rats’ social behaviour were collected several decades ago when the research emphasis was on aggression and competition^21,22^. More recent research has greatly contributed to our understanding of play behaviour^23^, pro-social tendencies^24^, and cooperation^25^, although the typical housing conditions of rats in laboratories and the social manipulations used to induce these behaviours, such as isolating individuals from their groups or applying food colorant to their fur^26,27^, limit the generalization of the knowledge gained through these studies to other social contexts.

Thus, the nature and stability of differentiated associations in rats remains understudied. To our knowledge, only one study has investigated the occurrence of differentiated relationships with group members in captive female rats, concluding that although rats show considerable levels of cooperation, they associate randomly and lack stable relationships^28^. However, this study included only a few observations of each group (i.e., a total of two observation sessions of one hour each per group) while rats were in either altered (i.e., their home cage with all hiding possibilities removed) or new environments (i.e., an unfamiliar big arena), which could have affected their association patterns. More importantly, since social relationships are defined as the outcome of a series of social interactions between individuals over time, and observation time affects the probability of observing individuals interacting, social preferences are likely to be detected only by more comprehensive studies of behaviour. Furthermore, this previous study focused on female rats and it is currently unknown whether male rats display similar association patterns. Here we examine the occurrence of differentiated associations in group living male rats by applying network analysis on social interactions recorded over a three-month observation period. Social network analysis is a powerful and flexible tool for analysing association patterns in animals that takes into account not only direct (dyadic) interaction (as methods quantifying variation in pairwise association indices do), but also indirect connections between individuals^29,30^. We then examine whether any observed patterns of social preferences are consistent across time and whether dominance rank plays a role in determining association patterns.

## Results

### Distribution of social relationships

**Figure 1:**
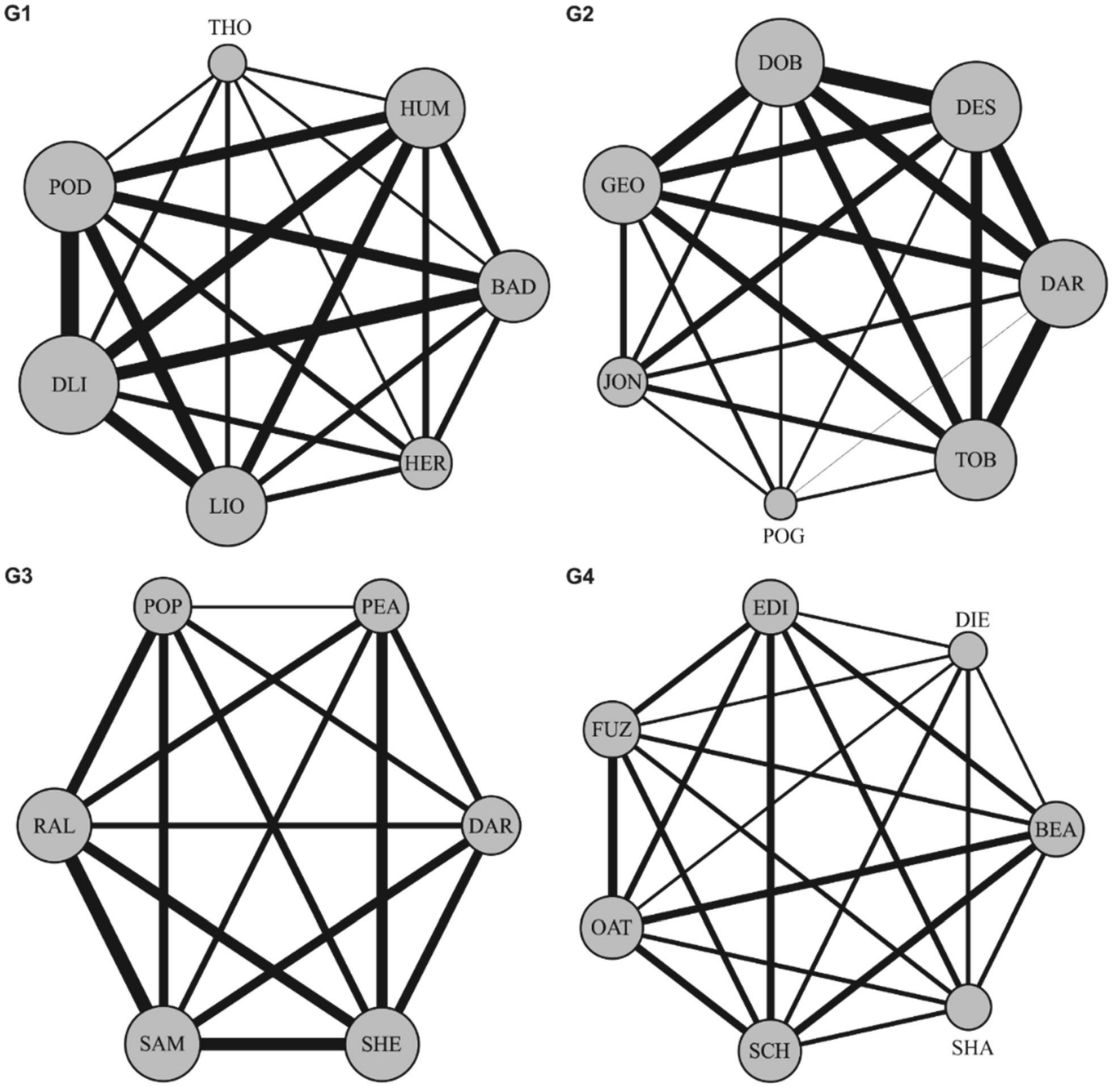
Social network diagrams for study groups (G1-G4). Nodes represent individual rats and their size is proportional to an individual’s network strength. Edge thickness represents the association index (SRI) between individuals. The three letter codes indicate individual rats’ names.

Twenty-seven male Fancy rats (i.e., domesticated rats with a variety of fur patterns typically kept as companion animals) living in four groups (G1 = 7, G2 = 7, G3 = 6, and G4 = 7) were the subjects of the study. When testing whether the observed association patterns could have arisen by chance, given spatial proximities, we found that the overall gregariousness (i.e. the average network strength) of all groups was significantly different from the average network strength of random association (G1*:* mean strength = 3.359, mean random strength ± SD: 2.774 ± 0.155, *P*_*rand*_ = 0.0001; G2*:* mean strength = 3.354, mean random strength ± SD: 2.752 ± 0.154, *P*_*rand*_ = 0.0001; G3*:* mean strength = 3.057, mean random strength ± SD: 2.414 ± 0.149, *P*_*rand*_ = 0.0002, G4*:* mean strength = 2.479, mean random strength ± SD: 1.965 ± 0.118, *P*_*rand*_ = 0.0005, Fig. 1), suggesting that patterns of association between individuals were not random (see Table S1 for results using only body contact data).

Furthermore, the analyses also show that the observed network coefficient of variation (CV) was significantly different from the random networks CV in all groups but one (i.e. G1, G2, G3), suggesting that individuals in these groups had significantly more preferred/avoided relationships than expected at random (permutation tests, G1: CV = 0.462, mean random CV ± SD 0.439 ± 0.012, *P*_*rand*_ = 0.026; G2: CV = 0.498, mean random CV ± SD 0.461 ± 0.015, *P*_*rand*_ = 0.008; G3: CV = 0.287, mean random CV ± SD 0.236 ± 0.016, *P*_*rand*_ = 0.001). However, in one of the groups (G4), CV from the observed and random networks were not significantly different, indicating that for this group the inter-individual association patterns did not differ from random (permutation test, CV = 0.277, mean random CV ± SD 0.264 ± 0.015, *P*_*rand*_ = 0.216) (see Table S1 for results using only body contact data).

### Consistency of association patterns over time

When analysing the consistency of the social preferences we found that for two of the study groups (G1, G2) there was a significant positive correlation between the association network of the first and second observation periods (Mantel test; G1: *z* = 1.87, *P* = 0.040; G2: *z* = 1.96, *P* = 0.013), whereas no relationship was found for the other two groups (Mantel test; G3: *z* = 1.45, *P* = 0.129; G4: *z* = 0.89, *P* = 0.928).

### Association patterns and dominance relationships

Both observed Landau’s corrected index (h’) and the directional consistency index (DCI) values were non-significant in all cases (Table 1), indicating that rats in this population do not form linear dominance hierarchies. The h’ index ranged between 0.31 and 0.68 and the DCI between 0.41 and 0.75. Furthermore, there was a very high proportion of two-way interactions in all groups (Table 1). When testing the effect of dominance scores on social associations we found that across groups, individual network strength was not significantly related to individual dominance scores (coefficient ± SE: 0.016 ± 0.022, *t* = 0.759, *P*_*rand*_ = 0.442), suggesting that the observed association patterns are not driven by the social status of individuals.

**Table 1:** Results from the Matman test for linearity of dominance hierarchies for the four study groups of rats.

Group	G1	G2	G3	G4
Matrix total	75	106	85	215
Landau’s corrected index (h′)	0.678	0.446	0.343	0.643
Directional consistency index	0.466	0.755	0.412	0.6
% of unknown relationships	19.05	0	6.67	0
% of one-way relationships	42.86	57.14	26.67	28.57
% of two-way relationships	38.10	42.86	66.67	71.43
% tied relationships	4.76	4.76	13.33	0
Improved linearity test (R-tailed probability)	0.103	0.284	0.641	0.110

## Discussion

Using a network approach, we quantified association patterns in groups of male rats over a three-month period. Our findings revealed that associations between rats were not randomly distributed and that most individuals have significantly more preferred/avoided associates than expected by chance, indicating that when kept in all-male groups, male rats can form differentiated social relationships. Evidence from a range of species shows that animals living in groups do not associate randomly with their group mates, which has been linked to individuals’ survival and reproductive performance [e.g. 3]. Multiple mechanisms can guide partner selection, with factors such as sex or kinship greatly influencing the distribution of social associations in many species^4^. For instance, in female philopatric species, females tend to associate preferentially with their maternal kin^5^. Since rats are able to recognise kin^31^, it is possible that rats’ association patterns may depend on the kinship relationship between group mates. Kinship information was not available for the study groups and therefore we were not able to explore this possibility. Future studies examining kinships and other possible mechanisms underlying social preferences in rats are therefore warranted.

The occurrence of non-random association patterns in rats is consistent with work in other species that has used quantitative measures of individuals’ behaviour. For instance, birds^15^, fish^32^, bats^12^, or primates^5^ are known to have preferred associates within their groups. Social preferences outside reproductive contexts are also found in some rodent species, such as house mice^7^ or striped mice (*Rhabdomys pumilio*)^33^. To our knowledge, our study is the first showing that male rats can also form non-random associations when living in all-male groups. However, we do not know whether these differentiated associations were driven mainly by preference towards certain individuals, which would suggest the occurrence of close social bonds in rats, or primarily by avoidance, leading to others moving into proximity. Therefore, further work is needed to determine the underlying drivers of these observed social differentiations.

Our findings, however, contrast with results from a previous study examining in-group social preferences in female wild-type Norway rats, which did not detect preferential associations in all-female groups^28^. There is extensive literature reporting sex differences in sociability and social behaviour in many mammal species, and how these sex differences are influenced not only by hormones, but also by developmental processes and prior social experiences^34^. Thus, it is possible that male rats, but not female rats, establish non-random associations with group mates. However, female rodents are in general more social and less resilient to isolation than males^35^, and females from other rodent species do exhibit differentiated social relationships, which significantly impact their fitness^7^. Thus, given the occurrence of differentiated associations in group-living rodents, including male rats, and their relevant fitness consequences, it is possible that female rats may have the potential for forming preferential associations when given the choice between several social partners. However, these associations might be of a looser nature than those found in female-bonded species (e.g., some primates) and therefore difficult to readily detect without sufficient sampling effort and the application of powerful analytical tools, such as the ones used in this study. A further possibility is that the tendency of forming differentiated social relationships in rats depends on their strain. Although the overall affiliative, agonistic and sexual repertoire among different strains is similar, there is much variation in the frequency, degree of asymmetry and complexity of their behaviours, with wild-type strains typically showing lower levels of social tolerance and higher levels of agonistic behaviours^36–38^. Thus, it is possible that social preferences are formed in some strains (e.g., fancy rats, this study), but not in others (e.g., wild-type rats^28^). It would be important that future research explore this possibility by investigating the occurrence of preferred associations in different rat strains bred and housed under similar conditions.

In addition to showing the presence of differentiated associations in groups of male rats, our results also suggest that these associations can be stable over time. In half of our study groups, there was a significant positive correlation between the association network of the first and second observation periods, indicating consistency of their social preferences, for a period of a few months at least. However, in the other two groups, we did not find any correlation between the association networks of the two observation periods, suggesting that although the number of their preferred/avoided associations might be similar between study periods, associations in these groups may be of a more opportunistic nature. Evidence accumulated over the last 15 years shows that in some group living species individuals are able to maintain stable social preferences over extended periods of time^39,40^. However, stability is not an essential or indispensable characteristic for social relationships to occur, nor to bring fitness benefits^41^. According to Hinde’s framework^10^, social relationships are not static entities, they are dynamic and can change over time due to both predictable (e.g., development) and unpredictable events (e.g., migration of individuals between groups). Some of the most common variables that modulate social preferences, such as significant changes in group composition or resource availability (e.g., food, hiding resting places), remained stable during our study period, but we did not measure other potential variables that could have affected relationship between group members (e.g., hierarchical changes^42^). Further studies exploring variables affecting relationships formation and maintenance over a longer period of time and on a larger sample size of male, female and mixed-sex groups of rats will further our understanding of the nature of differentiated associations and social dynamics in rats.

We also found that rats in this population did not form linear dominance hierarchies. In many social species, including rats, the dominance status of individuals is mainly determined by their fighting ability (e.g. red deer, *Cervus elaphus*,^43^; rats,^21^) and outcomes of dyadic interactions can be used to construct their dominance hierarchies. Although previous descriptions of dominance relations in male rats suggest clear and stable rank orders^44^, systematic analyses of the linearity of their dominance hierarchies are typically not reported and thus it is difficult to compare previous work with our findings. However, it should be noted that a lack of linearity in a dominance hierarchy does not imply lack of dominance relations between individuals. All dyads in a group can have clear dominance relations, and yet the overall hierarchy could be non-linear if it contains intransitive triads.

Analyses of dominance rank revealed that male rats’ associations were not influenced by individuals’ rank position, suggesting that dominance rank is not responsible for the observed pattern of social preferences. Social associations can be affected by dominance rank if, for instance, individuals prefer to associate with high-ranking partners who can potentially provide greater benefits than low-ranking individuals (e.g., access to monopolised resources). Association biases in favour of dominant individuals have been reported for a variety of species. For instance, hyenas associate more often with dominant individuals than with subordinate ones^45^, and primates from many species of cercopithecine monkeys tend to affiliate more often with high-ranking than low-ranking group mates^13^. Similarly, when mixed-sex groups of wild-type rats are kept in semi-naturalistic environments, adult males form clear dominance relationships with high-ranking males being frequently contacted by lower-ranking ones^21^. However, clear dominance relationships are not always observed in domesticated rat strains^46^ and males in our colony showed a high proportion of two-way interactions resulting in a lack of linearity in their dominance hierarchies. Therefore, it is possible that this lack of linearity in their dominance hierarchies reduced the attractiveness of higher-ranking individuals.

While our study focused only on all-male groups of rats, thus limiting the generalisation of our findings, our results show that rats have the potential of forming non-random associations with group mates. Our results can also have important implications for the management and welfare of captive rat populations, and in particular for those colonies where rats are housed in large same-sex groups, as is often the case with pet rats. Accumulated evidence (including this study) shows that group living animals often form non-random associations with fellow group members, which can generate strong benefits to individuals, from tolerance around limited resources^47^ to social thermoregulation^48^. More importantly for captive environments, social partners have also been shown to positively affect psychological, physiological, and behavioural functions, as well as to attenuate physiological stress^49–51^. Thus, providing individuals with appropriate social environments that enable them to exercise partner choice (i.e., more than one single partner) could positively affect their welfare. On the other hand, the disruption of preferred associations can also have negative consequences on individuals. In both humans and non-human animals, disruption of social relationships can lead to an increase of physiological and behavioural stress responses^52,53^. While the effect of the removal of individual rats from their social groups is known to affect social stability^54^, the welfare implications of the disruption of rats preferred social associations remains to be studied. A better understanding of rats’ sociality is increasingly necessary in lab animal husbandry, as more animals are housed in groups rather than individually. We suggest that our findings on differentiated relationships in rats are incorporated in future work addressing the welfare of captive rat populations.

Finally, the fact that male rats can have preferred/avoided partners in their groups has also important implications for research examining physiological, neurological or behavioural processes. For instance, familiar individuals (i.e., individuals co-habiting an enclosure for a period of time) have been found to mitigate conspecific’s stress responses more effectively than unfamiliar ones^55,56^, but this effect has not always been found^57,58^. Similarly, familiarity seems to influence how animals use social information in diverse species, including rats, with animals prioritizing information from familiar individuals in some studies^59,60^ but not in others^61,62^. It might be possible that variation in social preferences for familiar individuals could in part explain these contradictory results. We suggest that research addressing the role of familiarity on physiological, neurological or behavioural processes in rats consider including a finer measure of individual’s social preferences.

## Methods

### Ethical note

This study adheres to all legal requirements and guidelines of the U.K. government and to the ASAB/ABS guidelines for the Treatment of Animals in Behavioural Research and Teaching, and was carried out in compliance with the ARRIVE guidelines. The delegated authority of the University of Lincoln Research Ethics Committee approved this research (CoSREC374).

### Subjects and housing

Twenty-seven male Fancy rats (i.e., domesticated rats with a variety of fur patterns typically kept as companion animals) living in four groups (G1 = 7, G2 = 7, G3 = 6, and G4 = 7) were the subjects of the study. On arrival at the Animal Unit of the University at circa 6–8 weeks of age, rats were housed in groups (6–7 individuals) based on combining individuals with differing markings to aid identification and remained in their groups for their natural lifespan or until rehomed as companion animals. At the start of the study, rats were approximately 8 months old, ensuring that the social groups were well established. Each group was housed in Mid-West Critter Nation Animal Habitat cages (double units: 61 × 91 × 124 cm) with platforms at different heights, and several shelters, nesting material (i.e., shredded paper), and enrichment (e.g., a variety of toys, chewing material, ropes, climbing tubes). Room temperature was 21.0 ± 2.0 °C and natural light was provided from dimmed windows. Supplementary artificial light was used during husbandry activities (e.g., cleaning and feeding) if needed. Rats were scatter fed (Supreme Science Selective, supplemented with fresh vegetables at least once a week) and water was available ad libitum. Individuals were recognisable through distinguishing characteristics (i.e., size, and natural colour patterns), except in the case of five individuals who were marked using red-food dye applied to their backs (individuals THO, DLI (Group 1), DAR, JON (Group 2), SAM (Group 3), Fig. 1). In order to avoid any possible effect of dye application on rats’ behaviour, behavioural recording started at least 15 min after dye was applied. Red dye was chosen because rats do not see those wavelengths^63^.

### Behavioural measures

Behavioural data were collected from video recordings (SONY HDR-CX675 & HikVision DS-7600) between January and March 2018, with a total of 120 recording sessions of 30 min each; that is, 30 recordings per group, each recording taking place on a different day, thus totalling 30 recording days, which is estimated to be enough to detect the occurrence of non-random associations for moderately to highly socially differentiated networks^64^. Video recordings were taken while the rats were in their enclosures during day time (between 12:00 and 16:00) and outside feeding periods. Although rats have been described as being primarily nocturnal^65^, they can also be active during the day^37^, and the rats in this study were regularly handled and participated in behavioural and cognitive tests during the light period, including on days when behavioural observations were conducted. All rats participated in the same behavioural and cognitive tests, were tested individually, and none of the tests involved any social tasks. During recording sessions, the animals were left undisturbed for the entire session. Recordings were later used to conduct scan samples every 2 min of the state behaviours body contact (i.e., any body part of two individuals (except their tails) were in physical contact), and proximity (i.e., individuals were within one body length (circa 20 cm)). Body contact and proximity were only recorded if individuals were in the same level of their enclosure with no physical obstacles between them and were not exchanging any agonistic behaviour. All videos were coded by CAT. A subset (5%) of the videos was coded by an independent observer who was naïve to the aims of the study. Scores for social proximity (i.e., individuals in contact or in proximity) were highly correlated between the two coders (r = 0.89).

### Dominance

In order to assess dominance relationships between individuals, 5 min controlled observations were conducted in which a small amount of food (< 10% of their daily food intake) was given to each group prior to the evening feeding time. During the observations, the occurrence and direction of any agonistic interaction (including biting, wrestling, boxing, aggressive posturing, chasing, pinning, and fleeing, see^21^ for definitions), along with supplants (i.e., individual approaches another one and gains access over space or food without showing any aggressive behaviour and the other retreats without showing any submissive behaviour) were recorded using an all-occurrence sampling technique^66^. A total of 30 dominance observations per group were conducted (i.e., 150 min per group) during the study period, and only dyadic interactions were used to determine social dominance. For each interaction, the participant supplanting (i.e., gaining access to food or space), chasing, biting or pinning down their opponent was designated the winner, and the other individual the loser. If both individuals exhibited aggressive behaviours, then the conflict was considered undecided and no winner and loser designated. Dominance matrices were constructed using the total number of wins and losses (see Table S2).

### Data analysis

#### Distribution of social relationships

Analyses were conducted in R v. 3.4.4^67^. Social associations between individuals were calculated using social proximity data (i.e., individuals were in contact or in proximity) recorded during scan sampling. Non-directed weighted association networks for each group of rats were generated using the *asnipe* package^68^ for the total observation period. Associations between individuals were then calculated using the simple ratio index (SRI^69^) which estimates the probability of co-observing two individuals in social proximity versus being observed on their own. This association index is commonly used in network analysis when observations between individuals are rarely missed during data collection^64^, as is the case in the present study. Social network diagrams were produced to visualise interaction patterns between rats using the *igraph* package in R^70^.

In order to investigate if overall gregariousness in groups differed from random, average network strength (i.e., the average of the sum of the edge weights (SRI) of each node (individual)^64^) was calculated for all groups using the *igraph* package^70^ and compared to the average network strength of random association networks generated from 10,000 data-stream permutations, which sequentially swap associations between pairs of individuals observed at the same time point^29,68^. Additionally, to test if rats have more preferred/avoided relationships than expected at random (i.e., differentiated associations), the coefficient of variation (CV) of the SRIs between individuals of the observed association networks for each group was compared to the CV of the SRIs measured for random association networks generated as described before^29^. Significance for both measures was estimated by comparing the observed metric for each group (i.e., average network strength and CV) to the distribution of the same metric generated using the permutations^29^.

#### Consistency of association patterns over time

To examine the consistency of the association patterns over time, we built association networks for the first and second half of the observation period for each group (i.e., 240 sample points on each period) using the *get_network* function in the *asnipe* package (version 1.1.11^68^). We then ran Mantel tests using 10,000 permutations (*ape* package^71^) to test for an association between the networks of the first and second observation period for each group.

#### Association patterns and dominance relationships

In order to evaluate dominance relationships (cf.^72^) we carried out hierarchical rank order analysis^73^. Dominance networks of dyadic interactions were constructed using the total number of wins and losses and then analysed by means of MatMan (Noldus, v1.1). Hierarchies were estimated using both the de Landau’s index of linearity (h’)^73^ and the directional consistency index (DCI), since de Landau’s index provides inaccurately low linearity index values when the number of individuals or interaction frequencies are low^74^. Additionally, we calculated individual dominance scores using the David’s score method (DS)^75^, which is a type of cardinal rank measure based upon dyadic dominance proportions (i.e., individual’s proportions of wins and losses), taking into account the relative strengths of opponents^76^.

A linear mixed-effects model (using the *lmer* function of the *lme4* package^77^) was conducted to investigate if an individual’s network strength differed as a function of the individual dominance scores (DS). Group was included as a random effect. Significance was measured by comparing the t- statistic extracted from the output of the linear model fitted to the observed data with the t-statistic calculated from models fitted to randomly generated network data using 10,000 data-stream permutations using the *asnipe* package in R^78,79^.

## Supplementary Information

Supplementary Information.

## Data Availability

The datasets analysed during the current study are available at 10.24385/45818.
