# Supplementary material for: Non-random associations in group housed rats (Rattus norvegicus)
Source: Sci Rep. 2021 Jul 28;11:15349. doi: 10.1038/s41598-021-94608-4 (PMC8319288; doi:10.1038/s41598-021-94608-4)
Supplement: Supplementary file 1 — Supplementary Information. [file 41598_2021_94608_MOESM1_ESM.pdf]

SUPPLEMENTATY INFORMATION

Non-random associations in group housed rats (*Rattus norvegicus*)

Leanne Proops[a], Camille A Troisi[b,c], Tanja K Kleinhappel[b], Teresa Romero[b*]

[a] Centre for Comparative and Evolutionary Psychology, Department of Psychology, University of Portsmouth, UK

[b] School of Life Sciences, College of Sciences, University of Lincoln, UK

[c] Department of Experimental Psychology, Ghent University, Belgium

*Corresponding author: Teresa Romero. School of Life Sciences, College of Sciences, University of Lincoln, UK

tromero@lincoln.ac.uk

	Average network strength			Coefficient of variation (CV)		
**Group**	**Strength**	**Random strength (±SD)**	P_{rand}	**CV**	**Random CV (±SD)**	P_{rand}
G1	3.317	2.717 ± 0.158	<0.0001	0.493	0.467 ± 0.013	0.007
G2	3.287	2.643 ± 0.154	<0.0001	0.528	0.492 ± 0.013	0.002
G3	3.016	2.364 ± 0.148	<0.0001	0.297	0.243 ± 0.017	0.002
G4	2.369	1.840 ± 0.107	<0.0001	0.329	0.307 ± 0.017	0.088

Table S1. ***Distribution of Social Relationships.*** Average network strength and average coefficient of variation for networks constructed using only body contact data

G1

	BAD	DLI	HER	HUM	LIO	POD	THO
BAD	-	0	1	3	7	4	3
DLI	0	-	0	0	0	0	0
HER	0	0	-	2	3	0	1
HUM	2	0	0	-	5	3	7
LIO	0	3	2	5	-	0	1
POD	2	1	1	5	2	-	4
THO	0	0	0	4	3	1	-

G2

	DAR	DES	DOB	GEO	JON	POG	TOB
DAR	-	9	3	2	2	9	0
DES	0	-	1	2	3	16	3
DOB	1	0	-	1	0	3	4
GEO	0	4	0	-	1	1	2
JON	0	5	4	2	-	17	1
POG	1	0	2	0	0	-	3
TOB	1	0	1	0	1	1	-

G3

	DAR	PEA	POP	RAL	SAM	SHE
DAR	-	3	9	0	1	5
PEA	3	-	5	3	1	1
POP	3	5	-	1	1	4
RAL	3	8	3	-	0	0
SAM	0	3	3	1	-	0
SHE	4	10	3	2	0	-

G4

	BEA	DIE	EDI	FUZ	OAT	SCH	SHA
BEA	-	2	2	0	6	7	5
DIE	7	-	5	0	3	0	0
EDI	7	7	-	1	11	2	2
FUZ	3	12	9	-	9	9	2
OAT	14	14	4	1	-	9	6
SCH	2	12	11	0	5	-	3
SHA	7	2	11	0	2	1	-

Table S2. ***Dominance matrices of the four study groups***. Rows represent the winners and the columns the losers in dyadic agonistic encounters (see Methods sections for full description). The three letter codes indicate individual rats' names.
